# Prognostic Value of Hepatic T1 Mapping in Patients with Takotsubo Syndrome

**DOI:** 10.3390/jcm15052050

**Published:** 2026-03-07

**Authors:** Riccardo Cau, Alessandro Pinna, Marco Francone, Alessia Pepe, Amalia Lupi, Emilio Quaia, Maria Francesca Marchetti, Roberta Montisci, Rodrigo Salgado, Luca Saba

**Affiliations:** 1 Department of Radiology, Azienda Ospedaliero Universitaria (A.O.U.), di Cagliari–Polo di Monserrato s.s. 554 Monserrato, 09045 Cagliari, Italy; 2 Faculty of Medicine and Surgery, University of Cagliari, 09042 Cagliari, Italy; 3 Department of Medical-Surgical Sciences and Traslational Medicine, School of Medicine and Psychology, Sapienza-University of Rome, Sant’Andrea Hospital, 00189 La Spezia, Italy; 4 Department of Medicine, University of Padova, 35128 Padova, Italy; 5 Department of Cardiology, Azienda Ospedaliero Universitaria (A.O.U.), di Cagliari–Polo di Monserrato s.s. 554 Monserrato, 09045 Cagliari, Italy; 6 Department of Radiology, Universitair Ziekenhuis Antwerpen, 2650 Edegem, Belgium

**Keywords:** takotsubo syndrome, CMR, T1 mapping, liver, outcomes

## Abstract

**Objective:** Takotsubo syndrome (TTS) is an acute form of heart failure characterized by transient left ventricular systolic dysfunction. Given the complex cardiohepatic interactions observed in heart failure, this study aimed to evaluate the prognostic significance of hepatic T1 mapping in patients with TS. **Materials and Methods:** In this retrospective pilot study, cardiovascular magnetic resonance (CMR) including hepatic T1 mapping was performed in 66 consecutive patients with TTS (60 females; mean age 70.96 ± 10.11 years). The median duration of long-term follow-up was 7 months (interquartile range, 2–16 months). The primary endpoint was a composite of out-of-hospital all-cause mortality and major cardiovascular or cerebrovascular adverse events, including heart failure hospitalization, TTS recurrence, and ischemic stroke. **Results:** During the median follow-up period of 7 months, 12 (18%) patients experienced the primary endpoint. Kaplan–Meier analysis revealed a significantly lower event-free survival in patients with higher hepatic T1 values (log-rank, *p* = 0.001). In multivariable Cox regression analysis, hepatic T1 mapping emerged as an independent predictor of adverse outcomes (HR 1.010; 95% CI 1.002–1.017, *p* = 0.010). **Conclusions:** Elevated hepatic T1 mapping values were independently associated with an increased risk of adverse cardiovascular events during follow-up. Incorporating hepatic T1 mapping into the clinical evaluation of patients with TTS may improve risk stratification and support more personalized management strategies.

## 1. Introduction

Takotsubo syndrome (TTS) is an acute, reversible form of heart failure characterized by transient left ventricular systolic dysfunction [1,2,3,4,5,6]. TTS is a potentially serious acute heart failure condition rather than a benign entity, given the considerable rate of in-hospital complications and long-term adverse cardiovascular events, underscoring the need for improved risk stratification strategies [7,8,9,10,11,12,13,14,15]. Emerging evidence suggests that TTS is not limited to myocardial dysfunction but also has a multi-organ dimension, reflecting a systemic response to acute stress [16,17,18,19]. Among these extracardiac manifestations, the cardiohepatic axis has gained increasing attention. The heart and liver are closely interconnected: cardiac dysfunction may induce hepatic impairment through venous congestion and reduced perfusion, whereas hepatic dysfunction can exacerbate systemic inflammation and worsen cardiac outcomes [20,21,22,23,24]. Impairment of this cardiohepatic axis has been identified as an independent predictor of cardiovascular events and mortality across several cardiovascular diseases [25,26,27]. Cardiovascular magnetic resonance (CMR) is the reference non-invasive imaging modality for the comprehensive evaluation of cardiac structure, function, and tissue characteristics in TTS, offering both diagnostic and prognostic information [28,29,30,31,32,33]. T1 relaxation time has proven useful in characterizing both myocardial and hepatic tissue properties, providing insight into passive hepatic congestion during acute cardiac dysfunction [25,34,35]. Notably, the liver’s anatomical proximity to the heart allows its assessment using standard CMR mapping sequences, without the need for additional acquisitions. In a recent study by Cau et al., TTS patients demonstrated significantly higher hepatic T1 values compared with age-, sex-, and risk factor-matched controls, suggesting that hepatic T1 mapping may serve as a sensitive imaging biomarker of cardiohepatic interaction. Furthermore, hepatic T1 values were independently associated with right ventricular longitudinal strain and N-terminal pro–B-type natriuretic peptide (NT-proBNP), both well-established markers of adverse prognosis in TTS [36].

Building on these preliminary findings, the present study aimed to investigate the prognostic impact of hepatic T1 mapping in TTS patients, evaluating its potential role in identifying patients at increased risk of adverse cardiovascular events during follow-up.

## 2. Materials and Methods

### 2.1. Study Population

This retrospective, single-center observational study included all consecutive patients diagnosed with TTS between 3 March 2017 and 30 November 2024. A total of 66 patients met the diagnostic criteria established by the Heart Failure Association of the European Society of Cardiology (ESC) [37].

The diagnosis of TTS was established based on the presence of regional LV wall motion abnormalities extending beyond a single epicardial coronary territory, typically occurring after an identifiable emotional or physical stressor, in the absence of culprit obstructive coronary artery disease on invasive coronary angiography (ICA). Additional elements supporting the diagnosis included newly developed electrocardiographic changes, elevated natriuretic peptide concentrations with disproportionately mild troponin release, and normalization of LV systolic performance at follow-up evaluation.

Patients were excluded if they were younger than 18 years, had a previous history of myocardial infarction, established cardiomyopathy, had evidence suggestive of permanent myocardial damage, had inadequate hepatic coverage on T1 mapping sequences, or documented hepatic or biliary disorders.

Baseline cardiovascular risk factors were extracted from electronic medical records. Hypertension was defined as resting systolic blood pressure ≥140 mmHg and/or diastolic blood pressure ≥90 mmHg documented on at least two separate measurements, or ongoing antihypertensive treatment [38]. Participants were categorized as current smokers or non-smokers; individuals who had stopped smoking more than six months before study inclusion were classified as non-smokers. Dyslipidemia was defined by LDL cholesterol levels ≥ 130 mg/dL (≥3.4 mmol/L) and/or non-HDL cholesterol ≥160 mg/dL (≥4.1 mmol/L) [39]. Cholesterol levels were measured according to the standard in-house laboratory protocol. Diabetes mellitus was diagnosed according to World Health Organization criteria or based on a previously established diagnosis of type 2 diabetes [40]. Obesity was defined as a body mass index (BMI) exceeding 30 kg/m^2^, in accordance with international guidelines [41].

**Figure 1:**
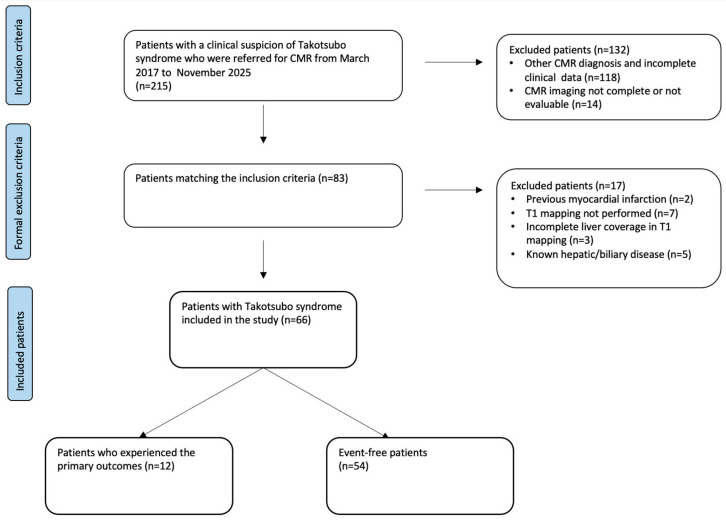
Flowchart of the enrolled patients.

The study protocol received approval from the local Institutional Review Board. Due to the retrospective design, the requirement for written informed consent was waived. A detailed diagram illustrating patient selection and the application of inclusion and exclusion criteria is presented in Figure 1.

### 2.2. CMR Acquisition

CMR examinations were performed at 4.2 ± 2.9 days after symptom onset (median = 4 days; range = 1–9 days) using a 1.5 T Philips Achieva dStream scanner system (Philips Healthcare, Best, Amsterdam, The Netherlands).

The imaging protocol included short- and long-axis cine sequences, T2-STIR, T1 and T2 mapping on three short-axis slices (basal, mid-ventricular, and apical), and late gadolinium enhancement (LGE) imaging in short- and long-axis views.

Balanced steady-state free precession (bSSFP) cine sequences were acquired during breath hold with retrospective ECG gating (TE = 1.7 ms; TR = 3.4 ms; flip angle = 45°; slice thickness = 8 mm). Long-axis views comprised two-, three-, and four-chamber projections. A contiguous short-axis stack (10–15 slices) was obtained from the atrioventricular ring to the apex to ensure full biventricular coverage.

T2-STIR images were obtained using triple inversion recovery T2-weighted pulse sequence (TR = 2 RR, TE ≈ 70 ms; flip-angle: 45°, section thickness = 8 mm, FOV 300 × 300 mm^2^). Images were acquired in both long-axis and short-axis planes to provide complete ventricular visualization.

Native T1 mapping was obtained prior to contrast administration using an ECG-triggered modified Look-Locker inversion recovery (MOLLI) 5s(3s)3s scheme in three short-axis slices (basal, mid-ventricular, and apical levels) during a single breath hold (TE = 1.12 ms; TR = 2.5 ms; flip angle = 35°; FOV = 300 × 300 mm).

T2 mapping was also performed before contrast injection on the same three short-axis levels using a black-blood-prepared, ECG-synchronized multi-echo spin-echo sequence acquired in breath-hold conditions.

First-pass perfusion imaging was acquired immediately after contrast injection (gadobutrol, Gadovist; Bayer Healthcare, Berlin, Germany) at a dose of 0.15 mL/kg body weight. Three representative short-axis slices (basal, mid, and apical) were acquired using an inversion recovery-prepared echo-planar imaging sequence (prepulse delay = 100 ms; TE = 1.1 ms; TR = 1.8 ms; flip angle = 30°; field of view = 300 × 300 mm^2^) with image acquisition spanning approximately 80 cardiac cycles.

LGE imaging was performed 10–12 min after contrast injection using the same gadobutrol dose (0.15 mL/kg). A phase-sensitive inversion recovery (PSIR) sequence was used for scar assessment in both short- and long-axis views.

### 2.3. CMR Image Post-Processing

**Figure 2:**
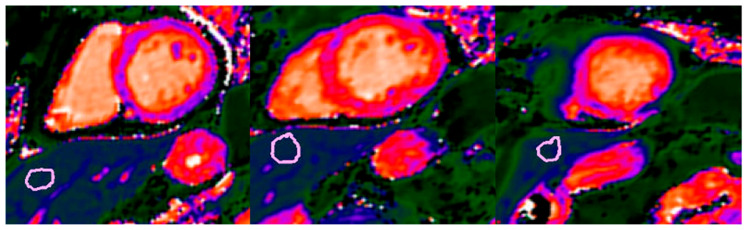
Representative example of hepatic T1 mapping quantification in a patient with Takotsubo syndrome. Hepatic T1 values were obtained by averaging measurements from three carefully selected regions of interest (ROIs, purple circles) within the liver parenchyma, each approximately 2 cm^2^ in area. To ensure accurate sampling of hepatic tissue, areas containing blood vessels and intrahepatic fat were meticulously excluded. This was achieved by cross-referencing the T1 mapping images with corresponding steady-state free precession cine images, allowing precise delineation of parenchymal regions while minimizing contamination from non-parenchymal structures.

Image analysis was performed using a commercially available software package (cvi42, version 6.0; Circle Cardiovascular Imaging Inc., Calgary, AB, Canada) for the assessment of myocardial function, volumes, strain, T1 and T2 mapping, and late gadolinium enhancement. Hepatic T1 values were measured by a single experienced observer (>10 years in cardiovascular imaging). Three circular regions of interest (approximately 2 cm^2^ each) were manually positioned within homogeneous liver parenchyma, carefully excluding visible vessels and focal fat. The average value across the three ROIs was used for analysis, as previously described [25,36]. ROIs were cross-checked on corresponding bSSFP images to ensure accurate exclusion of vascular structures and adjacent tissues (Figure 2).

Biventricular endocardial and epicardial borders were manually traced on short-axis cine images at end-diastole and end-systole. Papillary muscles and trabeculations were considered part of the ventricular cavity and excluded from myocardial mass calculations. Volumes (end-diastolic and end-systolic) and stroke volume were calculated using the Simpson’s summation-of-disks method. Ejection fraction was derived as (EDV − ESV)/EDV × 100. Ventricular mass values were indexed to body surface area.

LGE was evaluated qualitatively and quantitatively using PSIR images and interpreted according to the standardized 17-segment American Heart Association model. For quantitative assessment, a reference ROI was placed in visually normal myocardium without enhancement. Areas were considered enhanced if signal intensity exceeded 2 standard deviations above the reference region. LGE burden was expressed as a percentage of total LV mass.

### 2.4. Definition of Outcomes

Patients were followed up with after CMR through scheduled clinical evaluations and systematic review of hospital documentation.

The primary endpoint consisted of a composite endpoint including all-cause mortality and major cardiovascular or cerebrovascular events, specifically heart failure hospitalization, TS recurrence, and ischemic stroke.

Pulmonary edema was defined as the presence of respiratory distress and pulmonary rales due to pulmonary congestion, confirmed by chest radiography, respiratory failure (hypoxaemia-hypercapnia), tachypnoea (>25 breaths/min), and increased work of breathing [42].

TTS recurrence was defined as the development of new transient LV wall motion abnormalities in the absence of obstructive coronary disease, occurring after documented recovery from the initial episode [43].

Ischemic stroke was defined as cerebral infarction secondary to embolic or thrombotic occlusion of a major intracranial artery [44].

### 2.5. Statistical Analysis

Continuous variables were expressed as mean ± standard deviation (SD) or median and interquartile range (IQR), as appropriate, whereas categorical variables were presented as frequencies and percentages. Normality was assessed using the Kolmogorov–Smirnov test. Continuous variables were compared using Welch’s t-test when appropriate. Categorical data were analyzed using chi-square or Fisher’s exact test.

Univariable Cox proportional hazards regression was applied to identify predictors of the primary endpoint. CMR variables showing statistical significance (*p* < 0.05) in univariable analyses were subsequently entered into multivariable Cox regression models, adjusting for all variables significant at the univariable level.

Event-free survival was examined using Kaplan–Meier curves stratified according to hepatic T1 values. Patients were dichotomized based on the optimal cut-off value derived from receiver operating characteristic (ROC) curve analysis using Youden’s index. The optimal threshold for hepatic T1 mapping (490 ms) was identified as shown in Supplemental Figure S1. Subgroup comparisons were performed using the log-rank test.

All statistical tests were two-sided, and a *p*-value < 0.05 was considered statistically significant. Statistical analyses were conducted using JASP (version 0.96.0).

## 3. Results

### 3.1. Patient Population

During the inclusion period, a total of 66 patients with TTS were enrolled after applying the inclusion and exclusion criteria. Baseline characteristics of the study population are summarized in Table 1.

The mean age of the study population was 70.96 ± 10.11 years, and 6 patients (9%) were male. Over a median follow-up of 7 months (interquartile range [2,3,4,5,6,7,8,9,10,11,12,13,14,15,16]), 12 patients (18.2%) experienced the primary endpoint, including 5 hospitalizations for heart failure (7.5%), 1 recurrence of TS (1.5%), 5 deaths (7.6%), and 1 ischemic stroke (1.5%). The remaining 54 patients (81.8%) completed follow-up without events. No significant differences were observed in age (69.39 ± 10.98 vs. 71.27 ± 8.91 years; *p* = 0.656) or sex distribution (18.2% vs. 7.3% male; *p* = 0.261) between patients who experienced events and those who did not. Similarly, there were no significant differences in troponin T or NT-proBNP levels between the two groups.

Regarding cardiovascular risk factors, hypertension, diabetes mellitus, smoking, family history of coronary artery disease, and obesity were comparable between groups. Conversely, dyslipidemia was significantly less frequent among patients who experienced events (27.3% vs. 67.3%; *p* = 0.028). Dyspnea at presentation was more common in patients with subsequent events (45.4% vs. 20%; *p* = 0.046), while other clinical features, including chest pain, duration of hospitalization, and comorbidities, did not differ significantly.

**Figure 3:**
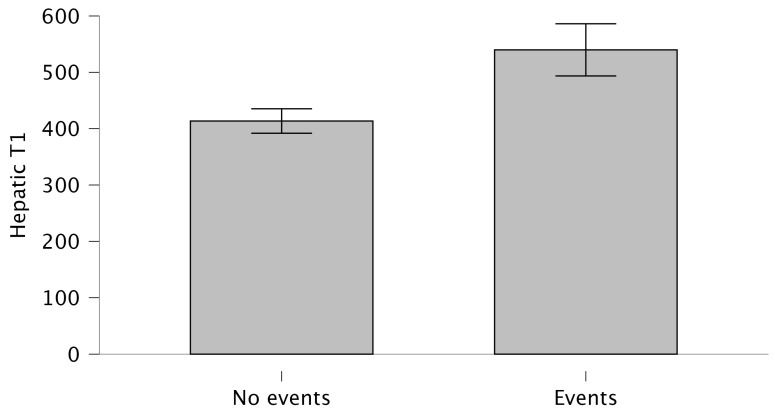
Box plots comparing patients with and without cerebrovascular events. Patients who experienced cerebrovascular events showed higher hepatic T1 value.

In terms of CMR findings, there were no significant differences in left or right ventricular volumes or systolic function between the two groups. Patterns of wall motion abnormality were similar across groups, with apical ballooning representing the predominant form (81.8% in both groups). The presence and extent of LGE, as well as myocardial T1 values, did not differ significantly. Hepatic T1 mapping values were markedly higher in patients who experienced adverse events compared with those without (539.87 ± 68.92 ms vs. 413.57 ± 80.28 ms; *p* = 0.001), as shown in Figure 3.

### 3.2. Associations of Hepatic T1 with Primary Outcomes

Variables associated with adverse events, as identified by univariable Cox proportional hazards regression analysis, are presented in Table 2.

Univariable analysis demonstrated that smoking (HR 4.831 [95% CI 1.220–19.123], *p* = 0.025), psychiatric disease (HR 5.185; 95% CI 1.069–25.131, *p* = 0.041), presence of intracardiac thrombus (HR 5.700; 95% CI 2.502–7.359, *p* = 0.003), and hepatic T1 mapping (HR 1.009; 95% CI 1.004–1.017, *p* = 0.001) were significantly associated with a higher rate of primary outcomes. On multivariable Cox regression analysis (Table 3), smoking (HR 3.687; 95% CI 1.124–6.658, *p* = 0.021), presence of intracardiac thrombus (HR 5.112; 95% CI 2.076–10.006, *p* = 0.007), and hepatic T1 mapping (HR 1.010; 95% CI 1.002–1.017, *p* = 0.010) remained statistically significant independent predictors of the primary outcomes. Specifically, a 1 ms increase in hepatic T1 value was associated with an approximate 1% increase in the risk of adverse events. To address the potential heterogeneity of the composite endpoint, a sensitivity analysis was performed restricting the outcome to all-cause death and major cardiovascular events, defined as recurrent TTS and hospitalization for heart failure. The association between hepatic T1 values and adverse events remained statistically significant (HR 1.013; 95% CI 1.005–1.021; *p* = 0.002).

**Figure 4:**
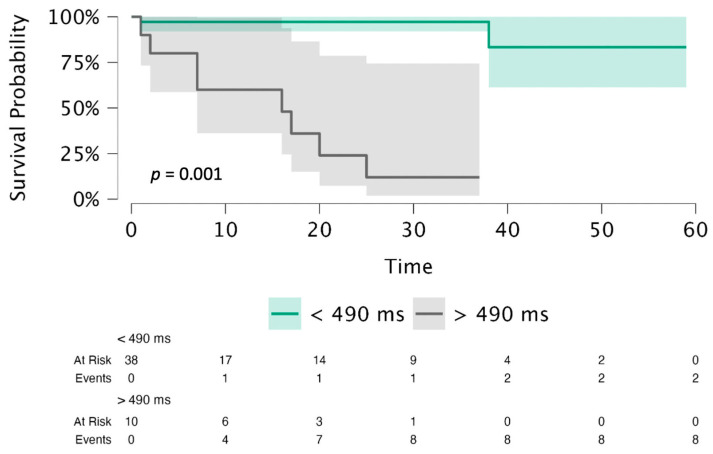
Kaplan–Meier curves of event-free survival according to hepatic T1 mapping. Patients discharged with hepatic T1 mapping <490 ms exhibited significantly higher survival free from adverse outcomes compared to those with hepatic T1 mapping > 490 ms.

Kaplan–Meier analysis demonstrated significantly lower event-free survival in patients with hepatic T1 mapping >490 ms, with higher rates of the primary outcome (log-rank *p* = 0.001; Figure 4), compared with TS patients with hepatic T1 mapping <490 ms.

## 4. Discussion

This study investigated the prognostic significance of hepatic T1 mapping in patients with TTS. Despite substantial progress in understanding TS, a considerable proportion of patients still experience significant morbidity and mortality, underscoring the need to identify novel predictors of outcome. Our findings demonstrate that elevated hepatic T1 values are independently associated with an increased risk of adverse cardiovascular outcomes during follow-up. Specifically, each 1 ms increase in hepatic T1 value corresponded to an approximate 1% increase in event risk.

The cardio-hepatic axis represents a dynamic and bidirectional interaction between the heart and the liver, in which alterations in cardiac function can lead to hepatic congestion, while hepatic dysfunction may, in turn, exacerbate systemic inflammation, oxidative stress, and neurohormonal activation—further impairing myocardial performance [20,23,25,36]. This pathophysiological interdependence has been recognized as a key determinant of outcomes across the spectrum of acute and chronic heart failure syndromes [20,23,25,36]. Hepatic dysfunction may therefore not only reflect the severity of cardiac compromise but may actively contribute to disease progression and adverse cardiovascular events through inflammatory and metabolic pathways [20,23,25,36].

In this context, hepatic tissue changes may provide valuable prognostic information, reflecting the degree of circulatory dysfunction and multi-organ involvement [25,36,45].

To the best of our knowledge, this is the first study investigating liver relaxometry as a prognostic imaging marker of the cardio-hepatic interaction in patients with TTS.

Our findings are consistent with the growing body of literature linking hepatic T1 mapping to adverse cardiovascular outcomes across various cardiac conditions. Guo et al. reported that higher hepatic native T1 values were independently associated with increased mortality and heart failure hospitalization in patients with pulmonary arterial hypertension [46]. Similarly, Kremer et al. demonstrated that hepatic T1-time correlated strongly with invasive hemodynamic parameters, including right ventricular diastolic stiffness and pulmonary vascular resistance, and predicted clinical worsening in pulmonary hypertension [47]. More recently, Bergamaschi et al. showed that hepatic T1 mapping reflected right ventricular involvement and was associated with higher NT-proBNP levels and increased rates of heart failure rehospitalization after acute myocardial infarction [25]. In addition, Mascherbauer et al. found that elevated hepatic T1-time was independently predictive of cardiovascular mortality and morbidity in a large cohort of patients undergoing CMR, irrespective of left or right ventricular function [26].

During the acute phase of TTS, transient left ventricular systolic dysfunction may lead to acute heart failure, initiating a cascade of systemic consequences that extend beyond the myocardium. The resulting reduction in cardiac output and concomitant rise in venous pressures promote tissue hypoperfusion and venous congestion, ultimately impairing hepatic perfusion. Consequently, even transient disturbances in cardiac function can disrupt hepatic oxygenation and nutrient delivery, leading to hepatocellular injury and interstitial changes. Importantly, hepatic impairment may worsen cardiovascular prognosis by amplifying systemic inflammatory responses and metabolic dysregulation, thereby promoting myocardial vulnerability and maladaptive remodeling [20,23,26,45,48].

### 4.1. Clinical Implications

The present findings highlight the potential clinical value of hepatic T1 mapping as a novel, non-invasive biomarker for risk stratification in patients with Takotsubo syndrome. Incorporating hepatic T1 assessment into routine CMR protocols could provide complementary information beyond traditional cardiac parameters, capturing the systemic effects of acute cardiac dysfunction and the degree of multiorgan involvement. Moreover, because hepatic T1 mapping can be derived from parametric sequences routinely acquired for myocardial tissue characterization, it offers additional prognostic insight without extending scan time or requiring contrast administration.

Elevated hepatic T1 values may help identify patients at higher risk of adverse cardiovascular outcomes, prompting closer clinical surveillance, optimization of medical therapy, and tailored follow-up strategies.

### 4.2. Limitations

This study is subject to several limitations. First, the retrospective design inherently limited the availability and completeness of certain clinical and laboratory data, as not all variables were systematically collected in every patient. Second, the relatively small sample size and limited number of adverse events may have increased the risk of overfitting in multivariable analyses, thereby constraining the robustness of conclusions regarding the incremental prognostic value of the proposed models. Larger, prospective studies with longer follow up are warranted to validate and strengthen these findings.

It is also important to recognize that native hepatic T1 values are not specific to congestion alone and may be influenced by a range of underlying conditions, including fibrosis, inflammatory processes, or iron deposition. Because a comprehensive multiparametric liver assessment was not performed, the specificity of our findings for passive hepatic congestion cannot be definitively established. Moreover, we did not systematically assess morphological markers of systemic venous congestion, such as inferior vena cava or hepatic vein dimensions, which could have provided additional mechanistic insight into whether T1 elevation reflects backward failure and elevated venous pressures. Future investigations integrating multiparametric liver imaging with biochemical profiling will be essential to disentangle these mechanisms and better characterize the pathophysiological meaning of hepatic T1 alterations in TTS.

Additionally, T1 mapping values are known to vary across CMR vendors, field strengths, pulse sequences, and post-processing approaches. Although all examinations in the present study were performed using a consistent acquisition protocol within a single center, this inter-vendor and inter-scanner variability may limit the direct reproducibility and generalizability of absolute hepatic T1 cut-off values across different imaging platforms.

Lastly, despite excluding patients with overt hepatobiliary disease, we did not systematically assess hepatic steatosis or subclinical liver abnormalities, which might have influenced T1 measurements. Additionally, CMR scans were acquired at variable time points after symptom onset, potentially influencing hepatic T1 and other imaging parameters, as both hepatic congestion and myocardial injury can evolve dynamically during the acute phase of TTS. Future studies adopting standardized imaging protocols and incorporating serial multiparametric assessments may help address these potential confounders and clarify the temporal relationship between cardiac dysfunction and hepatic involvement.

## 5. Conclusions

In patients with TTS, elevated hepatic native T1 values were independently associated with a higher incidence of adverse cardiovascular events. These findings suggest that hepatic T1 mapping, obtained without additional contrast administration, may represent a novel and clinically useful CMR-derived parameter for risk assessment in this population. Future studies are warranted to validate these findings and to further explore the pathophysiological mechanisms linking hepatic impairment and adverse cardiovascular events in this population.

## Figures and Tables

**Table 1:** Baseline and CMR characteristics of patients with Takotsubo syndrome.

Variables	Overall (n = 66)	Event (n = 12)	No Event (n = 54)	*p*-Value
**Age, mean (SD)**	70.96 ± 10.11	69.39 ± 10.98	71.27 ± 8.91	0.656
**Sex (Male), n (%)**	6 (9%)	2 (18.2%)	4 (7.3%)	0.261
**BPM, mean (SD)**	80.26 ± 16.41	75.40 ± 11.19	81.14 ± 17.12	0.308
**Troponin T, mean (SD)**	4473.71 ± 3988.76	4562.60 ± 3296.74	4457 ± 4137.55	0.579
**NTproBNP, mean (SD)**	1900.52 ± 3017.40	1574.50 ± 1317.51	1951.66 ± 3209.26	0.698
**Hypertension, n (%)**	37 (56%)	5 (45.5%)	32 (58.2%)	0.641
**Dyslipidemia, n (%)**	40 (60.6%)	3 (27.3%)	37 (67.3%)	**0.028**
**Obesity, n (%)**	9 (13.6%)	1(9.1%)	8 (14.5%)	0.715
**Smoke, n (%)**	10 (15.1%)	3 (27.3%)	7 (12.7%)	0.172
**Diabetes, n (%)**	9 (13.6%)	1 (9.1%)	8 (14.5%)	0.175
**CAD, n (%)**	9 (13.6%)	3 (27.3%)	6 (10.9%)	0.114
**COPD, n (%)**	5 (7.5%)	1 (9.1%)	4 (7.3%)	0.783
**Malignancies, n (%)**	12 (18.2%)	3 (27.3%)	9 (16.4%)	0.317
**Neurological disease, n (%)**	16 (24.2%)	4 (36.4%)	12 (21.8%)	0.228
**Psychiatric disease, n (%)**	8 (12.1%)	2 (18.2%)	6 (10.9%)	0.434
**Typical chest Pain, n (%)**	49 (74.2%)	7 (63.6%)	42 (76.4%)	0.679
**Dyspnea, n (%)**	16 (24.2%)	5 (45.4%)	11 (20%)	**0.046**
**Emotional Trigger, n (%)**	25 (37.9%)	3 (27.3%)	22 (40%)	0.288
**Physical Trigger, n (%)**	17 (25.8%)	3 (27.3%)	14 (25.4%)	0.923
**Duration of hospitalization, mean (SD)**	10.28 ± 7.12	11.50 ± 6.98	10.05 ± 7.19	0.341
**Apical ballooning, n (%)**	54 (81.8%)	9 (81.8%)	45 (81.8%)	0.607
**Midventricular ballooning, n (%)**	9 (13.6%)	1 (9.1%)	8 (14.5%)	0.701
**Basal ballooning, n (%)**	2 (3%)	1 (9.1%)	1 (1.8%)	0.187
**Focal Ballooning, n (%)**	5 (7.6%)	1 (9.1%)	4 (7.3%)	0.876
**LVEF, mean (SD)**	50.62 ± 10.26	47.33 ± 11.81	51.27 ± 9.91	0.228
**EDV/BSA LV, mean (SD)**	86.86 ± 40.12	91.31 ± 30.77	85.97 ± 41.91	0.348
**ESV/BSA LV, mean (SD)**	43.33 ± 25.79	50.28 ± 27.57	41.98 ± 24.94	0.242
**SV/BSA LV, mean (SD)**	43.19 ± 18.77	40.60 ± 6.77	43.70 ± 20.35	0.966
**RVEF, mean (SD)**	58.92 ± 5,65	58 ± 6.48	59.09 ± 5.52	0.692
**EDV/BSA RV, mean (SD)**	62.22 ± 23.79	64.26 ± 16.98	61.80 ± 25.25	0.267
**ESV/BSA RV, mean (SD)**	25.66 ± 10.75	27.22 ± 8.94	25.35 ± 11.12	0.286
**SV/BSA RV, mean (SD)**	36.76 ± 14.45	37.03 ± 9.85	36.12 ± 15.27	0.486
**Thrombus, n(%)**	4 (6%)	2 (18.2%)	2 (3.6%)	0.070
**T2 STIR, n (%)**	60 (90.9%)	9 (81.8%)	51 (92.7%)	0.261
**T2 STIR (segment involvement), mean (SD)**	5.9 ± 3.2	5.27 ± 3.34	6.03 ± 3.18	0.629
**LGE, n (%)**	20 (30.3)	2 (18.8%)	18 (32.7%)	0.347
**LGE (segment involvement), mean (SD)**	5.1 ± 2.6	3.2 ± 1.32	5.33 ± 2.72	0.189
**LGE 2SD, mean (SD)**	29.96 ± 12.57	24.10 ± 13.29	30.36 ± 12.77	0.601
**Hepatic T1 mapping, mean (SD)**	434.62 ± 91.99	539.87 ± 68.92	413.57 ± 80.28	0.001
**T1 mapping, mean (SD)**	1166.61 ± 94.50	1133.60 ± 58.48	1177.33 ± 102.87	0.199

Numbers in bold indicate a significant difference. Abbreviations: BPM, Beats Per Minute; BSA, body surface area; CAD, coronary artery disease; CMR, cardiovascular magnetic resonance; COPD, Chronic Obstructive Pulmonary Disease; EDV, end-diastolic volume; ESV, end-systolic volume; LGE, late gadolinium enhancement; LV, left ventricle; LVEF, left ventricular ejection fraction; RV, right ventricle; RVEF, right ventricular ejection fraction; SD, standard deviation; STIR, short tau inversion recovery; SV, stroke volume.

**Table 2:** Predictors of All-Cause Mortality and Out-of-Hospital Complications in Univariable Cox Regression Analysis.

Variables	HR	95% CI	*p*-Value
**Age**	0.982	[0.930, 1.038]	0.528
**Sex (Male)**	2.833	[0.594, 13.519]	0.191
**BPM**	0.956	[0.908, 1.008]	0.094
**Troponin T**	1.000	[1.000, 1.000]	0.846
**NTproBNP**	1.000	[1.000, 1.000]	0.548
**Hypertension**	0.941	[0.267, 3.309]	0.924
**Dyslipidemia**	0.309	[0.079, 1.205]	0.091
**Obesity**	1.402	[0.174, 11.283]	0.751
**Smoke**	4.831	[1.220, 19.123]	**0.025**
**Diabetes**	0.595	[0.074, 4.758]	0.624
**CAD**	2.328	[0.578, 9.366]	0.234
**COPD**	1.741	[0.214, 14.414]	0.604
**Malignancies**	1.731	[0.426, 7.037]	0.443
**Neurological disease**	2.408	[0.674, 8.604]	0.176
**Psychiatric disease**	5.185	[1.069, 25.131]	**0.041**
**Chest Pain**	0.760	[0.196, 2.947]	0.692
**Dyspnea**	3.325	[0.949, 11.649]	0.063
**Emotional Trigger**	0.411	[0.099, 1.703]	0.220
**Physical Trigger**	1.297	[0.324, 5.196]	0.713
**Duration of hospitalization**	1.030	[0.949, 1.121]	0.497
**Apical ballooning**	0.705	[0.088, 5.667]	0.743
**Midventricular ballooning**	0.298	[0.037, 2.376]	0.253
**Basal ballooning**	1.729	[0.214, 13.950]	0.607
**Focal Ballooning**	0.985	[0.118, 1.667]	0.943
**LVEF**	0.961	[0.904, 1.002]	0.202
**EDV/BSA LV**	0.992	[0.969, 1.015]	0.469
**ESV/BSA LV**	1.004	[0.967, 1.042]	0.837
**SV/BSA LV**	0.970	[0.930, 1.010]	0.147
**RVEF**	0.999	[0.898, 1.112]	0.992
**EDV/BSA RV**	0.982	[0.953, 1.012]	0.228
**ESV/BSA RV**	0.962	[0.901, 1.028]	0.254
**SV/BSA RV**	0.973	[0.927, 1.021]	0.264
**Thrombus**	5.700	[2.502, 7.359]	**0.003**
**T2 STIR**	0.467	[0.099, 2.028]	0.337
**T2 STIR (segment involvement)**	0.918	[0.758, 1.221]	0.401
**LGE**	0.770	[0.163, 3.648]	0.742
**LGE (segment involvement)**	0.886	[0.616, 1.275]	0.515
**LGE 2SD**	0.988	[0.867, 1.126]	0.858
**Hepatic T1 mapping**	1.009	[1.004, 1.017]	**0.001**
**T1 mapping**	0.993	[0.981, 1.005]	0.252

Numbers in bold indicate a significant difference. Abbreviations: BPM, Beats Per Minute; BSA, body surface area; CAD, coronary artery disease; CMR, cardiovascular magnetic resonance; COPD, Chronic Obstructive Pulmonary Disease; EDV, end-diastolic volume; ESV, end-systolic volume; LGE, late gadolinium enhancement; LV, left ventricle; LVEF, left ventricular ejection fraction; RV, right ventricle; RVEF, right ventricular ejection fraction; SD, standard deviation; STIR, short tau inversion recovery; SV, stroke volume.

**Table 3:** Predictors of All-Cause Mortality and Out-of-Hospital Complications in Multivariable Cox Regression Analysis.

Variables	HR	95% CI	*p*-Value
**Smoke**	3.687	[1.124, 6.658]	**0.021**
**Psychiatric disease**	5.182	[0.934, 8.151]	0.060
**Thrombus**	5.112	[2.076, 10.006]	**0.007**
**Hepatic T1 mapping**	1.010	[1.002, 1.017]	**0.010**

Numbers in bold indicate a significant difference.

## Data Availability

Data will be made available upon reasonable request to the corresponding author.

## Supplementary Materials

The following supporting information can be downloaded at https://www.mdpi.com/article/10.3390/jcm15052050/s1, Figure S1: Receiver operating characteristic (ROC) curves hepatic T1 mapping. ROC analysis identified optimal cut-off values of >490 ms for hepatic T1 mapping to discriminate between patients with and without adverse events. Cut-offs were derived using Youden’s index.

## Abbreviations

bSSFP	balanced steady-state free precession
CMR	cardiovascular magnetic resonance
ESC	European Society of Cardiology
LGE	late gadolinium enhancement
TIA	transient ischemic attack
TTS	Takotsubo syndrome

## References

[1] Ghadri J.-R., Wittstein I.S., Prasad A., Sharkey S., Dote K., Akashi Y.J., Cammann V.L., Crea F., Galiuto L., Desmet W. (2018). International Expert Consensus Document on Takotsubo Syndrome (Part I): Clinical Characteristics, Diagnostic Criteria, and Pathophysiology. Eur. Heart J..

[2] Ghadri J.-R., Wittstein I.S., Prasad A., Sharkey S., Dote K., Akashi Y.J., Cammann V.L., Crea F., Galiuto L., Desmet W. (2018). International Expert Consensus Document on Takotsubo Syndrome (Part II): Diagnostic Workup, Outcome, and Management. Eur. Heart J..

[3] Templin C., Ghadri J.R., Diekmann J., Napp L.C., Bataiosu D.R., Jaguszewski M., Cammann V.L., Sarcon A., Geyer V., Neumann C.A. (2015). Clinical Features and Outcomes of Takotsubo (Stress) Cardiomyopathy. N. Engl. J. Med..

[4] Cau R., Masala S., Manelli L., Porcu M., Scaglione M., D’Angelo T., Salgado R., Saba L. (2024). Cardiovascular Magnetic Resonance Imaging of Takotsubo Syndrome: Evolving Diagnostic and Prognostic Perspectives. Echocardiography.

[5] Ono R., Falcão L.M. (2016). Takotsubo cardiomyopathy systematic review: Pathophysiologic process, clinical presentation and diagnostic approach to Takotsubo cardiomyopathy. Int. J. Cardiol..

[6] Pelliccia F., Montalto E., Camici P.G. (2024). Recent highlights on Takotsubo syndrome. Int. J. Cardiol..

[7] Parodi G., Bellandi B., Del Pace S., Barchielli A., Zampini L., Velluzzi S., Carrabba N., Gensini G.F., Antoniucci D. (2011). Natural History of Tako-Tsubo Cardiomyopathy. Chest.

[8] Scudiero F., Arcari L., Cacciotti L., De Vito E., Marcucci R., Passaseo I., Limite L.R., Musumeci M.B., Autore C., Citro R. (2020). Prognostic relevance of GRACE risk score in Takotsubo syndrome. Eur. Heart J. Acute Cardiovasc. Care.

[9] Cau R., Muscogiuri G., Pisu F., Gatti M., Velthuis B., Loewe C., Cademartiri F., Pontone G., Montisci R., Guglielmo M. (2023). Exploring the Evolution in Prognostic Capability of Multisequence Cardiac Magnetic Resonance in Patients Affected by Takotsubo Cardiomyopathy Based on Machine Learning Analysis Design and Rationale of the EVOLUTION Study. J. Thorac. Imaging.

[10] Cau R., Palmisano A., Suri J.S., Pisu F., Esposito A., Saba L. (2024). Prognostic role of cardiovascular magnetic resonance in Takotsubo syndrome: A systematic review. Eur. J. Radiol..

[11] Santoro F., Gil I.J.N., Stiermaier T., El-Battrawy I., Guerra F., Novo G., Guastafierro F., Tarantino N., Novo S., Mariano E. (2019). Assessment of the German and Italian Stress Cardiomyopathy Score for Risk Stratification for In-hospital Complications in Patients With Takotsubo Syndrome. JAMA Cardiol..

[12] Ghadri J.R., Kato K., Cammann V.L., Gili S., Jurisic S., Di Vece D., Candreva A., Ding K.J., Micek J., Szawan K.A. (2018). Long-Term Prognosis of Patients With Takotsubo Syndrome. J. Am. Coll. Cardiol..

[13] El-Battrawy I., Santoro F., Stiermaier T., Möller C., Guastafierro F., Novo G., Novo S., Mariano E., Romeo F., Romeo F. (2021). Incidence and Clinical Impact of Right Ventricular Involvement (Biventricular Ballooning) in Takotsubo Syndrome: Results from the GEIST Registry. Chest.

[14] Morris N.A., Chen M.L., Adejumo O.L., Murthy S.B., Kamel H., Merkler A.E. (2020). Stroke Risk Following Takotsubo Cardiomyopathy. Neurohospitalist.

[15] Chong T.K., Chen J., Lyu L., Wei Y., Liu Y., Wu L., Tao Y., Jiang L., Sun Z., Li D. (2023). Clinical characteristics and outcome correlates of Chinese patients with takotsubo syndrome: Results from the first Chinese takotsubo syndrome registry. Int. J. Cardiol..

[16] Markousis-Mavrogenis G., Pepe A., Bacopoulou F., Lupi A., Quaia E., Chrousos G.P., Mavrogeni S.I. (2024). Combined Brain–Heart Imaging in Takotsubo Syndrome: Towards a Holistic Patient Assessment. J. Clin. Med..

[17] Cau R., Porcu M., Suri J.S., Cademartiri F., Mossa-Basha M., Saba L. (2025). Heart-Brain axis: Is microvascular dysfunction the link between stroke and Takotsubo syndrome?. Int. J. Cardiovasc. Imaging.

[18] Jung J.-M., Kim J.-G., Bin Kim J., Cho K.-H., Yu S., Oh K., Kim Y.-H., Choi J.-Y., Seo W.-K. (2016). Takotsubo-Like Myocardial Dysfunction in Ischemic Stroke. Stroke.

[19] Klein C., Hiestand T., Ghadri J.-R., Templin C., Jäncke L., Hänggi J. (2017). Takotsubo Syndrome – Predictable from brain imaging data. Sci. Rep..

[20] Capone F., Vacca A., Bidault G., Sarver D., Kaminska D., Strocchi S., Vidal-Puig A., Greco C.M., Lusis A.J., Schiattarella G.G. (2025). Decoding the Liver-Heart Axis in Cardiometabolic Diseases. Circ. Res..

[21] Jackson E., Dennis A., Alkhouri N., Samala N., Vuppalanchi R., Sanyal A.J., Muthiah M., Banerjee R., Banerjee A. (2025). Cardiac and liver impairment on multiorgan MRI and risk of major adverse cardiovascular and liver events. Nat. Med..

[22] Ford R.M., Book W., Spivey J.R. (2015). Liver disease related to the heart. Transplant. Rev..

[23] Xanthopoulos A., Starling R.C., Kitai T., Triposkiadis F. (2019). Heart Failure and Liver Disease: Cardiohepatic Interactions. JACC Heart Fail..

[24] Dichtl W., Vogel W., Dunst K.M., Grander W., Alber H.F., Frick M., Antretter H., Laufer G., Pachinger O., Polzl G. (2005). Cardiac hepatopathy before and after heart transplantation. Transpl. Int..

[25] Bergamaschi L., Arangalage D., Maurizi N., Pizzi C., Valgimigli M., Iglesias J.F., Landi A., Leo L.A., Eeckhout E., Schwitter J. (2024). Hepatic T1 mapping as a novel cardio-hepatic axis imaging biomarker early after ST-elevation myocardial infarction. Eur. Hear. J.-Cardiovasc. Imaging.

[26] Mascherbauer K., Donà C., Koschutnik M., Dannenberg V., Nitsche C., Duca F., Heitzinger G., Halavina K., Steinacher E., Kronberger C. (2022). Hepatic T1-Time Predicts Cardiovascular Risk in All-Comers Referred for Cardiovascular Magnetic Resonance: A Post-Hoc Analysis. Circ. Cardiovasc. Imaging.

[27] Huber A.T., Razakamanantsoa L., Lamy J., Giron A., Cluzel P., Kachenoura N., Redheuil A. (2020). Multiparametric Differentiation of Idiopathic Dilated Cardiomyopathy With and Without Congestive Heart Failure by Means of Cardiac and Hepatic T1-Weighted MRI Mapping. Am. J. Roentgenol..

[28] Bratis K. (2017). Cardiac Magnetic Resonance in Takotsubo Syndrome. Eur. Cardiol. Rev..

[29] Haghi D., Athanasiadis A., Papavassiliu T., Suselbeck T., Fluechter S., Mahrholdt H., Borggrefe M., Sechtem U. (2006). Right ventricular involvement in Takotsubo cardiomyopathy. Eur. Hear. J..

[30] Bossone E., Lyon A., Citro R., Athanasiadis A., Meimoun P., Parodi G., Cimarelli S., Omerovic E., Ferrara F., Limongelli G. (2014). Takotsubo cardiomyopathy: An integrated multi-imaging approach. Eur. Hear. J.-Cardiovasc. Imaging.

[31] Jensch P.-J., Stiermaier T., Eitel I. (2021). Takotsubo Syndrome—Is There a Need for CMR?. Curr. Heart Fail. Rep..

[32] Cau R., Loewe C., Cherchi V., Porcu M., Ciet P., Suri J.S., Saba L. (2022). Atrial Impairment as a Marker in Discriminating Between Takotsubo and Acute Myocarditis Using Cardiac Magnetic Resonance. J. Thorac. Imaging.

[33] Cau R., Bassareo P., Cademartiri F., Cadeddu C., Balestrieri A., Mannelli L., Suri J.S., Saba L. (2023). Epicardial fat volume assessed with cardiac magnetic resonance imaging in patients with Takotsubo cardiomyopathy. Eur. J. Radiol..

[34] Bogaert J., Claessen G., Dresselaers T., Masci P.G., Belge C., Delcroix M., Symons R. (2022). Magnetic resonance relaxometry of the liver - a new imaging biomarker to assess right heart failure in pulmonary hypertension. J. Heart Lung Transplant..

[35] Bogaert J., Symons R., Rafouli-Stergiou P., Droogné W., Dresselaers T., Masci P.G. (2021). Assessment of Right-Sided Heart Failure in Patients with Dilated Cardiomyopathy using Magnetic Resonance Relaxometry of the Liver. Am. J. Cardiol..

[36] Cau R., Pinna A., Marchetti M.F., Suri J.S., Montisci R., Saba L. (2025). Hepatic T1 Mapping in Takotsubo Syndrome: A Preliminary Imaging Insight into the Cardiohepatic Axis. Life.

[37] Lyon A.R., Bossone E., Schneider B., Sechtem U., Citro R., Underwood S.R., Sheppard M.N., Figtree G.A., Parodi G., Akashi Y.J. (2016). Current state of knowledge on Takotsubo syndrome: A Position Statement from the Taskforce on Takotsubo Syndrome of the Heart Failure Association of the European Society of Cardiology. Eur. J. Heart Fail..

[38] Unger T., Borghi C., Charchar F., Khan N.A., Poulter N.R., Prabhakaran D., Ramirez A., Schlaich M., Stergiou G.S., Tomaszewski M. (2020). 2020 International Society of Hypertension Global Hypertension Practice Guidelines. Hypertension.

[39] Civeira F., Arca M., Cenarro A., Hegele R.A. (2022). A mechanism-based operational definition and classification of hypercholesterolemia. J. Clin. Lipidol..

[40] World Health Organization (2006). Definition and Diagnosis of Diabetes Mellitus and Intermediate Hyperglycaemia: Report of a WHO/IDF Consultation.

[41] Flegal K.M., Carroll M.D., Kuczmarski R.J., Johnson C.L. (1998). Overweight and obesity in the United States: Prevalence and trends, 1960–1994. Int. J. Obes..

[42] Masip J., Peacok W.F., Arrigo M., Rossello X., Platz E., Cullen L., Mebazaa A., Price S., Bueno H., Di Somma S. (2022). Acute Heart Failure in the 2021 ESC Heart Failure Guidelines: A scientific statement from the Association for Acute CardioVascular Care (ACVC) of the European Society of Cardiology. Eur. Heart J. Acute Cardiovasc. Care.

[43] El-Battrawy I., Santoro F., Stiermaier T., Möller C., Guastafierro F., Novo G., Novo S., Mariano E., Romeo F., Romeo F. (2019). Incidence and Clinical Impact of Recurrent Takotsubo Syndrome: Results From the GEIST Registry. J. Am. Heart Assoc..

[44] Adams H.P., Bendixen B.H., Kappelle L.J., Biller J., Love B.B., Gordon D.L., Marsh E.E. (1993). Classification of subtype of acute ischemic stroke definitions for use in a multicenter clinical trial. Stroke.

[45] Santos R.R., Paiva M.S., Freitas P., Maltes S., Carvalho R., Pereira J.C., Domingues M., Santos A.C., Silva C., Guerreiro S. (2023). Hepatic t1 mapping: A new easily obtained biomarker for heart failure patients undergoing cardiac magnetic resonance. Eur. Heart J. Cardiovasc. Imaging.

[46] Guo J., Wang L., Wang J., Wan K., Gong C., Chen X., Guo J., Xu Y., He J., Yin L. (2022). Prognostic Value of Hepatic Native T1 and Extracellular Volume Fraction in Patients with Pulmonary Arterial Hypertension. J. Am. Heart Assoc..

[47] Kremer N., Roller F.C., Kremer S., Schäfer S., Kryvenko V., Rako Z.A., da Rocha B.R.B., Yogeswaran A., Seeger W., Guth S. (2024). Native hepatic T1-time as a non-invasive predictor of diastolic dysfunction and a monitoring tool for disease progression and treatment response in patients with pulmonary hypertension. Int. J. Cardiol..

[48] Kazour I., Serai S.D., Xanthakos S.A., Fleck R.J. (2018). Using T1 mapping in cardiovascular magnetic resonance to assess congestive hepatopathy. Abdom. Imaging.

