# Supplementary material for: Prognostic Value of Hepatic T1 Mapping in Patients with Takotsubo Syndrome
Source: J Clin Med. 2026 Mar 7;15(5):2050. doi: 10.3390/jcm15052050 (PMC12986253; doi:10.3390/jcm15052050)
Supplement: Supplementary file 1 [file jcm-15-02050-s001.zip › jcm-4172249-supplementary.pdf]

Supplemental Material

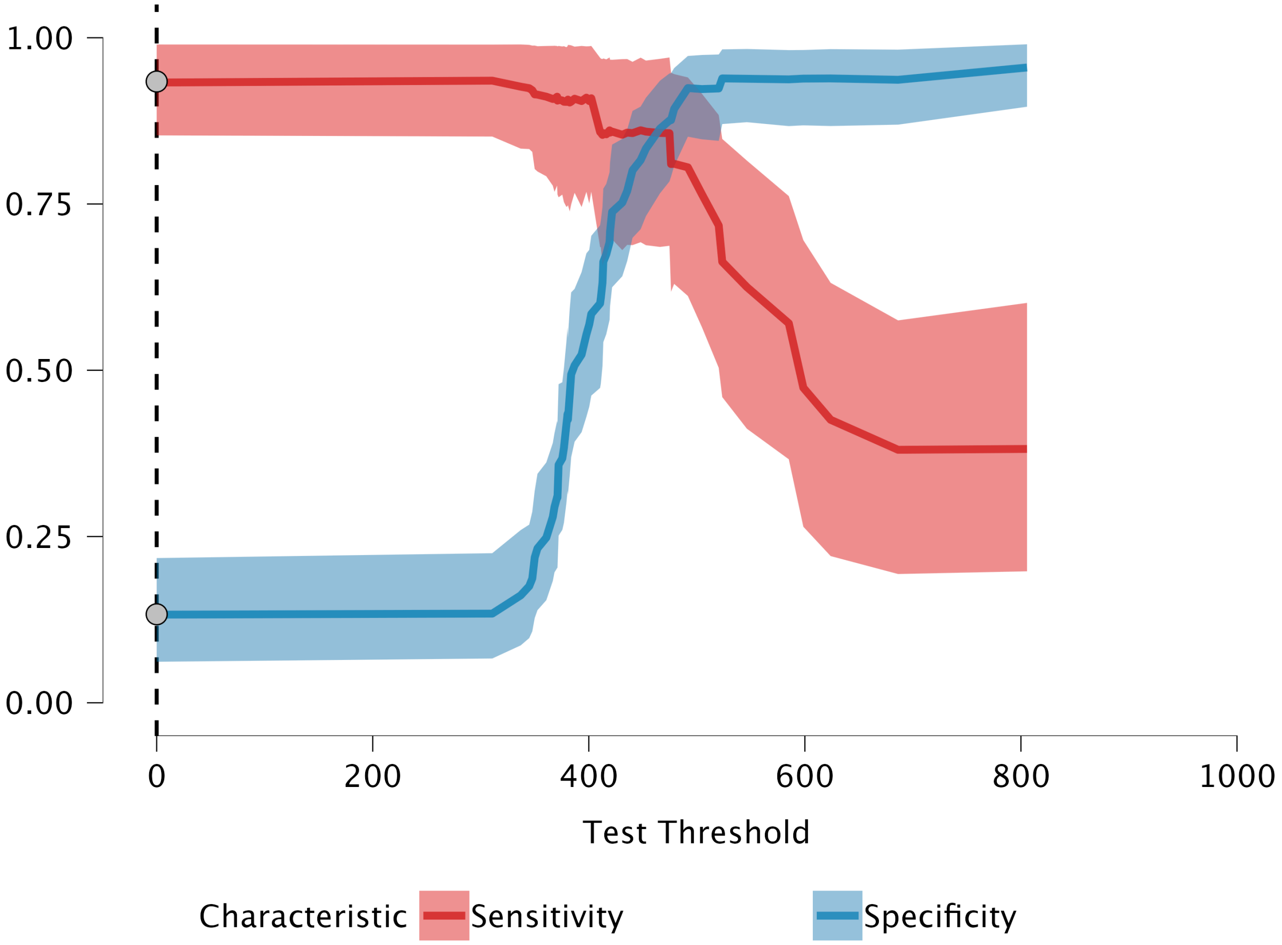

Supplemental Figure 1: Receiver operating characteristic (ROC) curves hepatic T1 mapping. ROC analysis identified optimal cut-off values of > 490 ms for hepatic T1 mapping to discriminate between patients with and without adverse events. Cut-offs were derived using Youden's index.
